# The musk chemical composition and microbiota of Chinese forest musk deer males

**DOI:** 10.1038/srep18975

**Published:** 2016-01-08

**Authors:** Diyan Li, Binlong Chen, Long Zhang, Uma Gaur, Tianyuan Ma, Hang Jie, Guijun Zhao, Nan Wu, Zhongxian Xu, Huailiang Xu, Yongfang Yao, Ting Lian, Xiaolan Fan, Deying Yang, Mingyao Yang, Qing Zhu, Jessica Satkoski Trask

**Affiliations:** 1 Farm Animal Genetic Resources Exploration and Innovation Key Laboratory of Sichuan Province, Sichuan Agricultural University, Chengdu, P.R. China, 61130; 2 Laboratory of Medicinal Animal, Chongqing Institute of Medicinal Plant Cultivation, Nanchuan, Chongqing, P.R. China, 408435; 3 Department of Anthropology and California National Primate Research Center, University of California, Davis, California

## Abstract

Male musk deer secrete musk from the musk gland located between their naval and genitals. Unmated male forest musk deer generate a greater amount of musk than mated males, potentially allowing them to attract a greater number of females. In this study, we used gas chromatography and mass spectrometry (GC/MS) to explore musk chemical composition of the musk pods of captive mated and unmated sexually mature Chinese forest musk deer and used next-generation sequencing to intensively survey the bacterial communities within them. Analysis of the chemical composition of the musk showed that unmated males have more muscone and cholesterol. Features of the musk16S rRNA gene showed that mated Chinese forest musk deer have both a greater Shannon diversity (*p* < 0.01) and a greater number of estimated operational taxonomic units than unmated ones; many bacterial genera were overrepresented in unmated Chinese forest musk deer males. Members of these genera might be involved in musk odor fermentation. PICRUSt analysis revealed that metabolic pathways such as aldosterone-regulated sodium reabsorption, metabolism of terpenoids and polyketides, flavone and flavonol biosynthesis, and isoflavonoid biosynthesis were enriched in the musk of unmated Chinese forest musk deer males.

The dwarf musk deer or Chinese forest musk deer (*Moschus berezovskii* Flerov) is an endangered artiodactyl species native to southern and central China and northernmost Vietnam^1^,^2^. Males and females are visibly very similar; neither carries antlers, but males are armed with two abnormally long canine teeth (Figure S1) in the upper jaw that are used in fights with rivals. On the abdomen, the sexually mature males carry the eponymous musk-pod^3^,^4^, into which a musk gland (Figure S2) secretes the musk, generally described as of the color and consistency of ‘moist gingerbread’ and which appears to serve for attracting the females^5^,^6^. Its secretion has a specific musk odor, and therefore the secretion’s chemical composition may be involved in chemical communication, potentially encoding information about sex and maturity^7^.

All animals harbor communities of microbes that profoundly affect their biology, often in beneficial ways^8^,^9^. It is also becoming obvious that symbiotic microbes can extend the host’s behavioral phenotypes in beneficial ways, including facilitating their feeding, antipredator, reproductive, and communicative behaviors^10^,^11^. The fermentation hypothesis for mammalian chemical communication posits that bacteria in the scent glands of mammals generate odorous metabolites used by their hosts for communication and that variation in host chemical signals is a product of underlying variation in the bacterial communities inhabiting the scent glands^12^. The musk owes its odor to a preponderance of a particular ketone, known as muscone^6^ which is generated by the preputial gland (Figure S2). Muscone is claimed to be the sexual attractant for musk deer females^13^. Previous studies have shown that myocardial ketone body metabolism can be regulated by the gut microbiota during nutrient deprivation^14^ giving us a hint that there may be interaction between the microbiome and compounds produced by the host, and potentially, musk pod microbiota and musk generation in Chinese forest musk deer. However, little is known about the composition of the microbiota of musk pod of male Chinese forest musk deer. In this study, we used next-generation sequencing to characterize and compare the bacterial communities in the musk pods of mated and unmated Chinese forest musk deer males (*Moschus berezovskii* Flerov).

## Results

### Collection of musk gland secretions (Musk) and sequencing

The fresh musk production records of captive Chinese forest musk deer males aged between 3.5–4.5 years were summarized in Table S1. The largest pouch obtained from an adult musk deer was from an unmated male (UM) (31.532 g), whilst the smallest, was only 0.422 g, in a mated male (MM). We found that musk production after mating was significantly less (*p* < 0.001) in mated than unmated deer. These 10 samples were collected and sent for sequencing. Sequence information of each sample was summarized in Table S1. The raw sequences of this study have been deposited in the Sequence Read Archive (accession number SRP059015).

### Musk chemical composition of mated and unmated males

**Figure 1:**
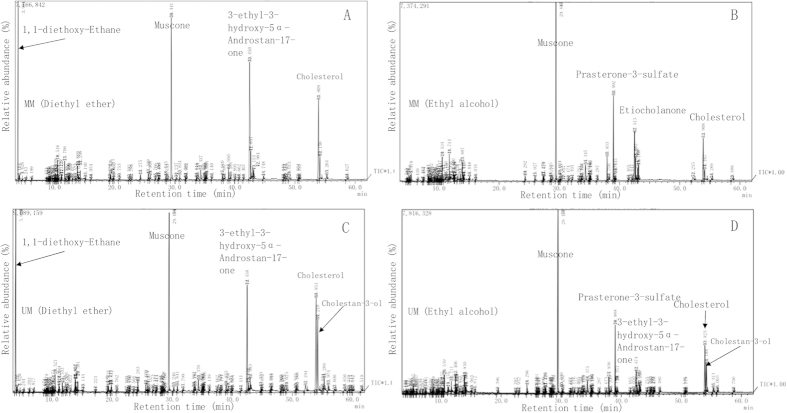
GC/MS chromatograms of the musk diethyl ether extraction of mated males (**A**) and unmated males (**C**), musk ethyl alcohol extraction of mated males (**B**) and UM (**D**). Only composition proportions greater than 5% were marked in all panels. The identities and relative abundances of the compounds are listed in Table 1.

Musk was separated by diethyl ether and absolute ethyl alcohol solution treatment. All potential musk chemical compositions of Chinese forest musk deer were listed in Table S2. The table only lists the top 15 peak areas of each sample. It was found that both solvents can extract 3-Methylcyclopentadecanone (muscone). Alkanes account for about 80% of the diethyl ether extract; while ethyl alcohol extract contains more steroid, aliphatic ester and cholesterol. After rapid conventional gas chromatography and mass spectrometry (GC/MS), the abundances of the main chemical constituents of the musk samples of mated and unmated Chinese forest musk deer males were determined (Fig. 1, Table 1 and Table S2). Percentages of muscone in unmated males are greater than mated males, 22.59% versus 18.55% and 35.76% versus 26.19% for diethyl ether and ethyl alcohol extract respectively. On the contrary, percentages of 1,1-diethoxy-Ethane (acetal) in MM are greater than UM (22.8 versus 19.6%).

### Features of the unmated and mated musk 16S rRNA gene

**Figure 2:**
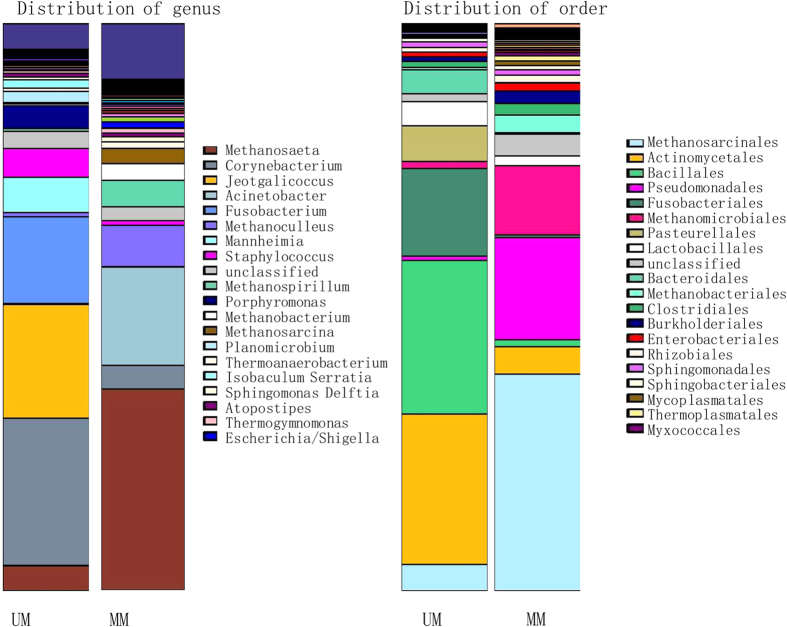
Composition variation of the unmated and mated microbiota analyzed employing 16S rRNA as biomarkers. Bars represent the relative abundance of bacterial taxa (genus and order). The top 20 abundant genus or orders are shown.

In this study, we characterized the musk microbiota of 5 mated male Chinese forest musk deer and compared them with that of 5 unmated males living in the same facility in Chongqing Institute of Medicinal Plant Cultivation. We performed multiplex pyrosequencing of the V3-V4 hyper-variable regions of 16S rRNA gene. 16S rRNA gene analysis using an operational taxonomic unit (OTU) definition of sharing ≥97% nucleotide sequence identity revealed that the bacterial communities in mated and unmated musk pods were markedly different but each was dominated by fermentative anaerobes. We generated a dataset consisting of 142,179 filtered, high-quality, classifiable 16S rRNA gene sequences with an average of 15572.80 ± 1166.56 (SE) and 12863.00 ± 2230.59 (SE) per sample in mated and unmated males. More than 95% of the sequences in all samples were found to belong to the five most populated bacterial phyla (namely Firmicutes, Actinobacteria, Fusobacteria, Proteobacteria and Bacteroidetes) and one archaea phyla (Euryarchaeota) (Table 2). For bacteria, Firmicutes, Actinobacteria, Fusobacteria and Bacteroidetes were present in higher proportions in UM than in MM deers’ microbiota (32.32% versus 5.45%, 26.63% versus 5.50%, 14.65% versus 0.47%, and 4.92% versus 1.72%, respectively). In contrast, Proteobacteria were more abundant in mated than in unmated males (29.44% versus 11.88%) (Table 2). For archaea, only Euryarchaeota were present in significantly higher proportions in MM than in UM deer (52.67% versus 7.67%, *p* < 0.01, Mann-Whitney U test). The majority of OTUs belonged to the division Firmicutes (167 ± 13.8) and Proteobacteria (351.6 ± 40.7) for UM and MM group, respectively (Table 2). The largest single OTU of UM and MM samples, is a member of the division Actinobacteria and genus *Corynebacterium* (26.02%) and the division Euryarchaeota and genus *Methanosaeta* (35.51%) respectively (Fig. 2).

### Diversity and richness of musk microbiota

**Figure 3:**
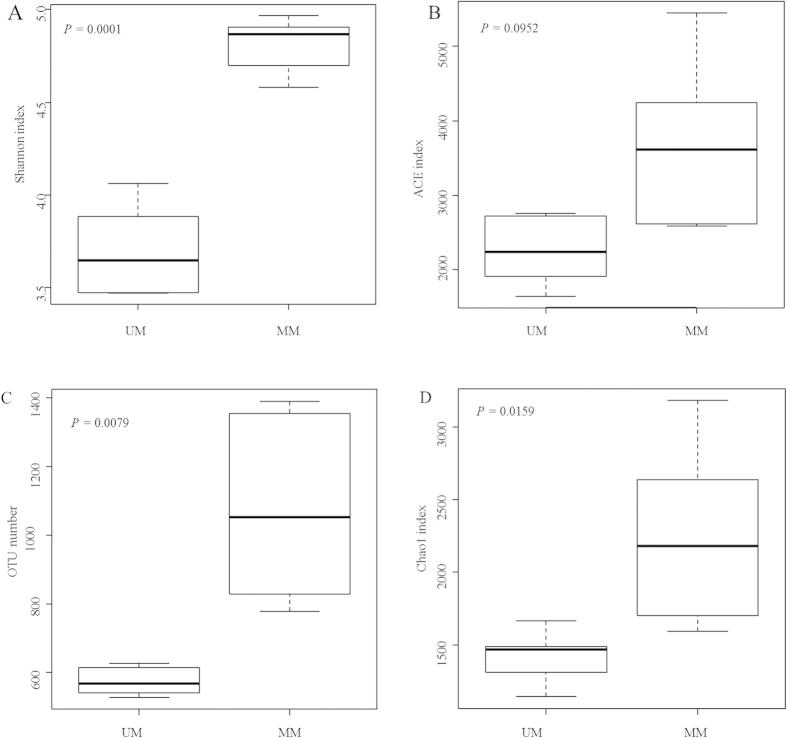
Variations in alpha diversity of the musk microbiota. (**A**,**B**) Comparisons of Shannon diversity (**A**), ACE diversity indices (**B**), the number of observed OTUs (**C**) and the number of estimated OTUs (Chao1) (**D**) between mated and unmated Chinese forest musk deer males by Mann-Whitney test. All alpha diversity metrics were calculated upon the rarified OTU subsets. In all panels, boxes represent the interquartile range (IQR) between the first and third quartiles. The lines inside boxes represent the median.

Four alpha diversity measures were calculated, including Shannon’s diversity index, ACE diversity index, observed species (observed OTUs), and Chao1 (estimated OTUs) (Fig. 3). Rarefaction curves using Shannon measure of alpha diversity were generated from musk samples obtained from both MM and UM deer (Figure S3). Each additional sample adds OTUs to the plot that were not observed in previous samples. The curve becomes asymptotic as the OTU number saturates, and each sample adds an increasingly smaller number of new OTUs, indicating adequate coverage for the environment being tested. We found significant difference in Shannon diversity (*p* < 0.01) between MM and UM musk samples (Fig. 3A). For community richness comparison, mated musk had significantly higher number of observed and estimated (Chao1) OTUs than unmated musk (*p* < 0.05, Fig. 3C,D and Figure S3). No significant differences in ACE diversity indices were observed between MM and UM musk samples (Fig. 3B).

**Figure 4:**
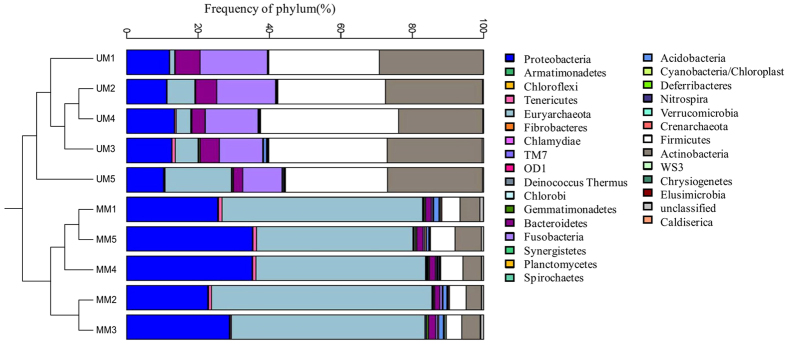
Clustering analysis of the evolution of the musk microbiotas of the mated and unmated Chinese forest musk deer males. Adjacent bar charts show the phylum level classification (as determined by the RDP classifier) for each of the sequences per sample.

Beta-diversity comparisons were performed using the Greengenes reference tree and computing phylogeny-based UniFrac distances^15^ between samples within QIIME. For exploring relationships between MM and UM musk samples’ phylogeny, both principal coordinate analysis (PCoA) on UniFrac distance matrices within QIIME and phylogenetic network analysis of community similarity^16^ were used to reconstruct networks from UniFrac distance matrices with SplitsTree4^17^ using the neighbour-netagglomerative method^18^. The relationship between the musk microbiotas of UM and MM were visualized by a dendrogram. Each branch on the tree represents one musk microbiota (Fig. 4). As expected, the neighbour-net network reconstructed from the unweighted UniFrac distance matrix on this data set still shows a clear pattern of clustering by their mating status (Fig. 4). Principal coordinate analyses (PCoA) were also used to examine the relationships between musk microbiota of MM and UM Chinese forest musk deer (Figure S4) and similar clustering patterns were observed. On the PCoA plot, each circle represents one gut microbiota. Consistent with the dendrograms, the musk microbiotas of the MM were distinct from those of UM. These results suggest that the microbiota may play a potential role in chemical composition of musk in Chinese forest musk deer.

### Specific musk bacteria of unmated Chinese forest musk deer males

The community structures of musk gland microbiota are significantly different between mated and unmated Chinese forest musk deer males. Members of the Corynebacterium, Fusobacterium, Jeotgalicoccus, Mannheimia and Planomicrobium are five of the several bacterial genera that were overrepresented in the UM microbiota. We did not find any evidence of the genus *Mannheimia* in the MM samples. The genus *Mannheimia* contains five species from the family Pasteurellaceae that have been recently reclassified^19^. The five named species within this genus are *M. haemolytica*, *M. varigena*, *M. glucosida*, *M. ruminalis*, and *M. granulomatis*^19^. Several unnamed taxa are also distinct from these named species^20^. The overrepresentation of this genus and other genus in unmated Chinese forest musk deer males indicates that members of this genus might be involved in fermentation.

**Figure 5:**
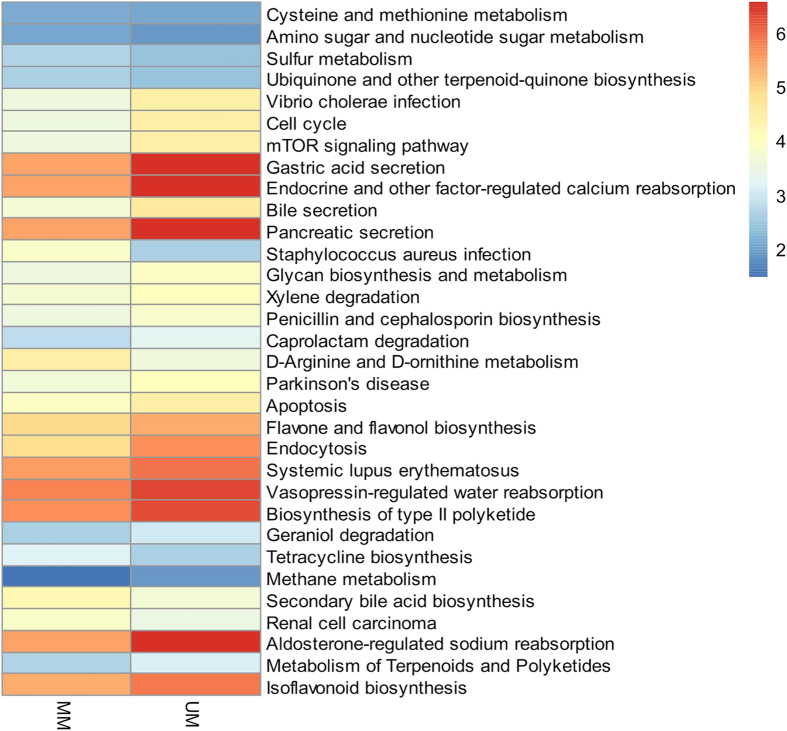
Predicted function of musk microbiota between musk of mated and unmated Chinese forest musk deer males. The gene copy numbers of five samples within the same sample group were pooled. Log10-transformed values for each functional gene (row) were used. KEGG pathway of the third level was shown in the heatmap. Statistical significance between the two groups of the gene distribution were performed using Mann-Whitney *U*-test with cutoffs of *p* < 0.01.

When the third-level KEGG pathways of these bacteria were examined, genes controlling metabolic pathways, including aldosterone-regulated sodium reabsorption, endocrine and other factor-regulated calcium reabsorption, xylene degradation, and aprolactam degradation (Fig. 5) were enriched in unmated males. Aldosterone-regulated sodium reabsorption and endocrine and other factor-regulated calcium reabsorption belong to the excretory system pathway; xylene degradation, and aprolactam degradation belong to the xenobiotics biodegradation and metabolism pathway, but the relationships of these pathways with musk odor is still unknown.

## Discussion

The primary biological significance of the musk gland secretion is to attract the females to males during the rutting season and to mark territory^7^. Muscone is the main active ingredient which is claimed to be the sexual attractant for musk deer females^13^. In this study we found that these unmated Chinese forest musk deer males secrete more musk than mated ones. By using gas chromatography and mass spectrometry we studied the chemical composition of mated and unmated males. The composition of musk from the Chinese forest musk deer preputial gland secretion coincides with data reported by Do *et al.* (1975)^21^ (see Table 1), with muscone accounting for 22.6% of secretion extract for Nepal musk deer^21^. It is known that acetalisation, or the organic reaction that involves the formation of an acetal, is a reversible reaction. One method of acetal formation is the nucleophilic addition of an alcohol to a ketone or an aldehyde. We found that muscone is more and acetal (1,1-diethoxy-Ethane) is less in unmated males, whilst muscone is less and acetal is more in mated males. Steroid compounds (cholestanol, cholesterol, and a number of the androstane derivatives) make up a large proportion of musk secretion components, which concurs with previously reported data^7^. However, these previous studies only found approximately ten kinds of chemical components, while we found more than 50 components in this study. Additional studies should be carried out to understand the relationship between mating status and musk chemical compounds.

All animals are inhabited by a mixed population of microbes, and contrary to popular belief, most microbes appear highly beneficial to their hosts. They are of critical importance in animal nutrition and immune defense, and can serve as crucial catalysts for the effective development and functioning of host tissues. Their contribution to host behavior is also becoming increasingly appreciated. It has been hypothesized that one way they do so is by producing the components of chemical signals that animals use to communicate. Our results demonstrate that the bacterial profiles of Chinese forest musk deer diverge between mated and unmated males. The production of musk is associated with its mating status. We propose the possibility that it isn’t the bacteria that are triggering musk production, but that after mating, the deer start to produce less musk through a physiological pathway, and only some bacterial strains can thrive in the new low-musk environment. We are also assuming that once the male mates, he produces little musk until the next rut, at which point he begins musk production and his microbiome changes to the unmated profile; given the anatomical position of the musk pod, is it also possible that the pod is getting “seeded” with novel bacteria from the female during the act of mating, further study is needed to confirm these hypothesis.

The microbiome plays an important role in human odour production and the number of certain microorganisms is strongly correlated with the intensity of the odour emitted^22^,^23^. Kevin *et al.*^12^, for example, showed that symbiotic bacteria underlie species-specific odors in both spotted and striped hyenas and further underlie sex and reproductive state-specific odors among spotted hyenas. The skin’s location at the interface with the outside world therefore makes it the most subject to environmental influences that will affect the microbiota^24^. Chinese forest musk deer males secrete a specific musk odor, and therefore the secretion’s chemical composition can be involved in chemical communication^7^, which appears to serve for attracting females^5^,^6^. Since mated Chinese forest musk deer males and unmated males produce various amount of musk, in combination with the fact that the musk pod has an opening to contact outside, it is easy to postulate that animals may share different musk microbiota. In this study, our results showed that mated Chinese forest musk deer males and unmated males harbor different musk microbiotas. The richness of musk microbial diversity in each sample was estimated by rarefaction curves, and ACE, Chao1 and Shannon indexes were calculated by MOTHUR (Fig. 2 and Figure S3). We concluded that OTU richness will increase with additional number of sequences. At least 1,732 and 3,061 OTUs were identified from UM and MM deer, respectively. Mated males showed diverse α-diversity than unmated males.

Despite some potential biases^25^, PICRUSt provides some insight into bacterial community functions in musk of Chinese forest musk deer. Muscone is claimed to be the sexual attractant for musk deer females^13^. In this study, we found many functional pathways related to ketone metabolism overrepresented in unmated Chinese forest musk deer males, for example, aldosterone-regulated sodium reabsorption, metabolism of terpenoids and polyketides, flavone and flavonol biosynthesis, and isoflavonoid biosynthesis pathways. It is important to note that the PICRUSt analysis should be considered speculative because there are also significant other unrelated pathways that appear such as Parkinson’s disease etc. The relevance of application of PICRUSt to predict bacterial activities in musk of Chinese forest musk deer needs further study. Further transcriptomics and metabolomics studies are desired to confirm these discoveries and improve our understanding of the bacterial functions in the musk of Chinese forest musk deer.

In summary, we characterized the musk microbiotas of the mated and unmated Chinese forest musk deer males and found that, according to the 16S rRNA based community structure analysis, the musk microbiota of these deer diverged based on their different mating condition. We also identified bacterial taxa differentially represented between the mated and unmated males. Further studies are needed to examine differential microbial roles in the hosts’ biochemical pathways and physiology.

## Materials and Methods

### Behavioral observations

The behavioral observations were carried out with 10 healthy Chinese forest musk deer males, kept in open-air yards, between 15^th^ Nov 2013 and 15^th^ Dec 2013, which coincided with the rutting season. Each male, along with 4 healthy females (3.5–4.5 years old with the similar body size and never mated before) was housed in individual breeding yards (about 500 m^2^), each yard have one big play ground and five small living rooms (about 8 m^2^ each) (Figure S5). Mating behavior was monitored by HIKVISION video cameras (Hangzhou, China), artificial observation by farmers, and the pregnancy of any females. After behavioral monitoring was complete, the farmers separated males and females with the least disturbance, as the females are easily frightened in the early stages of pregnancy. In order to prevent fighting among males, they were housed separately in 10 living rooms (12 m^2^), and there is a playground in front of those rooms (about 60 m^2^). Females were left in the original breeding yards. If we observed a male mating with females, they were classified as mated males. Meanwhile, if we did not observe any mating behavior or pregnancy they were classified as unmated males.

### Collection of musk gland secretions (Musk)

The mating season of the musk deer is from November to January^26^. The fresh musk specimens from 10 individuals (5 mated and 5 unmated) were obtained, weighed and recorded in early October (2014) (Table S1). They were all born in captivity and had not mated before. Their age range from 4.5 to 5.5 years old (Table S1). All samples of the musk pouches used for bacterial surveys were obtained directly from the musk gland scent pods of Chinese forest musk deer in the Chongqing Institute of Medicinal Plant Cultivation (Chongqing, China, altitude: 678m).

Musk deer were anesthetized with Telazol (4.5 mg/kg) delivered from a CO_2_ -powered darting rifle, especially while the musk deer was alone and lying down. A special spoon was used for musk collection from the pod. Musk samples were placed in sterile cryogenic vials, stored in liquid nitrogen, and transported to Sichuan Agricultural University, where they remained frozen at −80 °C until their bacterial profiles were determined. All animal experimentation were approved by the Institutional Animal Care and Use Committee of Sichuan Agricultural University under permit number DKY- S20143204. The methods were carried out in accordance with the approved guidelines.

### Investigation of main chemical components from preputial gland secretion

After microbial analysis, the rest of fresh musk was dried by exposure to phosphorus pentoxide (P_2_O_5_). We weighed the musk every 30 minutes until its weight ceased to change. Because a very small amount of musk remained for the mated group after bacterial DNA extraction and musk dehydration so we pooled three samples together (taking 0.07 gram of each sample, total 0.21 gram). After blending, each 0.1 gram of musk was dissolved in 2.5 ml absolute ethyl alcohol (purity > 99.7%) or 2.5 ml diethyl ether (purity > 99.5%), then extracted by ultrasonic for two hours. The dissolved samples were centrifuged at 13000g for 5 minutes and the liquid supernatant was used for chemical components analysis. The same method was applied for unmated (sample UM1, UM2 and UM3) males.

A Shimadzu GCMS-QP 2010 plus gas chromatograph (Shanghai, China), coupled to a Shimadzu QP 5000 mass spectrometer which was equipped with a split/split less injector and a DB5-MS column [(30 m × 0.25 mm i.d., 0.25 mm film thickness), (Agilent J&W, Agilent Technologies, CA, USA)] was used for the chromatographic analysis of the targeted compounds. The mass spectral data base NIST08.LIB was used to analyze the data. The confidence coefficient of the data above 80% was adopted.

### Chromatographic conditions

The injector and mass transfer-line temperatures were set at 280 °C and 290 °C, respectively. The column temperature was programmed at 60 °C for 5 min, increased to 150 °C at a rate of 10 °C/min and held for 5 min, then ramped at 5 °C/min to 280 °C (held for 5 min). The total run time was 58.5 min. The column pressure was set to 49.5 kPa and the column flow rate was 0.9 ml/min. The linear velocity was 34.7 cm/sec. The flux purge was 3.0 ml/min. The solvent cut time was set to 3.0 min. Sample injection was performed by autoinjection. The injection volume was 1.0 μL. The split ratio of diethyl ether extract and ethyl alcohol extract was set to 5:1 and 10:1, respectively. Helium (purity ≥ 99.99%) was used as the carrier gas at a flow rate of 1.0 ml/min. Selective ion monitoring (SIM) mode was adopted for the determination of the analytes.

### PCR and amplication

Total bacteria DNA was extracted from each 0.4 g musk samples by using MO BIO UltraClean fecal DNA kit (Carlsbad, CA, USA). Bacterial 16S rRNA genes in musk were PCR amplified using a set of two broadly conserved, degenerate primers targeting the V3–V4 variable regions of the 16S gene (341F: 5′–CCC TAC ACG ACG CTC TTC CGA TCT NXX XXX XXC CTA CGG GNG GCW GCA G–3′; 805R: 5′–GTG ACT GGA GTT CCT TGG CAC CCG AGA ATT CCA GAC TAC HVG GGT ATC TAA TCC–3′), the forward primer containing a 7-bp error-correcting barcode unique to each sample.

PCR reaction was conducted using a Mastercycler Gradient Thermal Cycler (Eppendorf) with the following condition: 94 °C for 3 min (initial dissociation), followed by 30 cycles of [94 °C for 30 s (denaturation), 60 °C for 45 s (annealing), and 72 °C for 1 min (extending)], followed by a final extension of 8 minutes at 72 °C. Negative non template controls were run for each barcoded primer pair to test for reagent contamination. Each sample was amplified and then pooled together for analysis. The PCR products were analyzed by gel electrophoresis and purified by using a PCR Purification kit (Sangon, Shanghai, China). Pyrosequencing was performed on an Illumina MiSeq 2 × 250 platform.

### Characterization of the similarities of the OTU of musk

QIIME^27^ pipeline was mostly used for data quality controls and analyses. Raw sequence reads were filtered to meet minimum and maximum length of 200 bp and 450 bp with no ambiguous base calls. Sequences that contained more than one ambiguous base call (N) and did not also have a complete barcode and primer at one end were eliminated. A read was discarded if it was identified as a putative chimera by UCHIME^28^. The sequences that passed the above procedure were denoised in order to correct for potential sequencing errors at 99% level using UCLUST^28^. Operational Taxonomic Unit (OTUs) were defined using UCLUST v1.1.579, which have been proven to generate satisfactory and comparable numbers of OTU^29^. The degree of similarity between sequences was defined as ≥97% as a threshold to obtain OTU identity at the species level. RDP classifier (version 2.10) software was used^30^ to classify the sequences according to the taxonomy proposed by Garrity *et al.*^31^, maintained at the Ribosomal Database Project (RDP 10 database, Update18). Richness, Shannon Index, ACE index, Chao1 and Coverage were included in Alpha diversity analysis by using MOTHUR^32^. Mann-Whitney U test was used for significance test. Weighted unifrac and unweighted unifrac metric distances, each with 10× subsampling, were calculated to obtain beta diversity index, which used phylogenetic tree to compute phylogenetic relatedness of the bacterial community between samples. These results were viewed by Principal Coordinate Analysis (PCoA)^15^. A two-sided Student’s t-test was used for significance test of beta diversity difference between mated and unmated musk deer. Metagenome functional content from 16S rRNA was predicted using PICRUSt (Phylogenetic Investigation of Communities by Reconstruction of Unobserved States)^33^. The R package ‘pheatmap’ was used for data analysis and plotting.

## Additional Information

**How to cite this article**: Li, D. *et al.* The musk chemical composition and microbiota of Chinese forest musk deer males. *Sci. Rep.* **6**, 18975; doi: 10.1038/srep18975 (2016).

## Supplementary Material

Supplementary Information

Supplementary Dataset 1

## Figures and Tables

**Table 1:** Chemical constituents of Chinese forest musk deer preputial gland secretion from ethyl alcohol and diethyl ether extraction (% of extract peak acreage).

Number	Chemical name	Molecular formula	CAS	Diethyl ether extract (%)		Ethyl alcohol extract (%)	
				MM	UM	MM	UM
1	1,1-diethoxy-Ethane (acetal)	C_6_H_14_O_2_	105-57-7	22.8	19.62	-	-
2	m-Cresol	C_7_H_8_O	108-39-4	1.1	0.73	1.84	1.41
3	6-Methylheptane-1,6-diol	C_8_H_18_O_2_	5392-57-4	0.86	0.35	1.83	0.76
4	1,2,6-Hexanetriol	C_6_H_14_O_3_	106-69-4	-	-	0.25	1.37
5	Benzeneacetic acid	C_8_H_8_O_2_	103-82-2	1.62	1	3.51	3.87
6	2,6,10,15-Tetramethylheptadecane	C_21_H_44_	54833-48-6	0.5	0.45	-	-
7	8-n-Hexylpentadecane	C_21_H_44_	13475-75-7	0.89	0.46	-	-
8	3-Methylcyclopentadecanone (Muscone)	C_16_H_30_O	541-91-3	18.55	22.59	26.19	35.76
9	Normuscone	C_15_H_28_O	502-72-7	0.39	0.65	0.63	1.04
10	14-Methyl-8-hexadecenal Z	C_17_H_32_O	60609-53-2	1.84	1.72	4.09	2.78
11	Olealdehyde	C_18_H_34_O	2423-10-1	0.38	0.28	1.54	0.91
12	12-Methyl-E,E-2,13-octadecadien-1-ol	C_19_H_36_O	0-00-0	0.41	0.71	0.11	0.08
13	13-Tetradecenal	C_14_H_26_O	85896-31-7	0.27	0.35	1.09	0.88
14	Doconexent	C_22_H_32_O_2_	6217-54-5	0.4	0.16	3.08	2.16
15	Prasterone-3-sulfate	C_19_H_28_O_5_S	651-48-9	0.91	0.37	12.11	9.02
16	Resibufogenin	C_24_H_32_O_4_	465-39-4	-	0.56	-	-
17	3α-Hydroxy-5β-androstan-17-one	C_19_H_30_O_2_	53-42-9	-	-	11.05	-
18	5α-Androstane-3α,17β-diol	C_19_H_32_O_2_	1852-53-5	3.37	-	3.2	0.79
19	Androsterone, trifluoroacetate	C_21_H_29_F_3_O_3_	0-00-0	0.97	0.87	2.12	1.27
20	Dihydroandrosterone	C_19_H_32_O_2_	0-00-0	-	-	2.34	0.73
21	3-ethyl-3-hydroxy-5α-Androstan-17-one	C_21_H_34_O_2_	57344-99-7	21.61	13.04	-	5.26
22	2-tert-Butyl-4,6-bis (3,5-di-tert-butyl-4-hydroxybenzyl)phenol	C_40_H_58_O_3_	134868-71-6	-	1.06	1.36	-
23	Cholesterol	C_27_H_46_O	57-88-5	11.44	12.42	8.73	9.37
24	Cholestan-3-ol	C_27_H_48_O	27409-41-2	3.1	8.13	2.44	5.55
25	Cholest-7-en-3β-ol	C_27_H_46_O	6036-58-4	0.62	1.48	0.47	0.97
26	4α-Methyl-5α-cholest-8(14)-en-3β-ol	C_28_H_48_O	62014-96-4	-	0.67	-	0.41

“−” indicate data are absent. The table only list top 15 extract peak acreage of each sample. 0-00-0 represent these chemicals which are currently not indexed in the CAS (Chemical Abstracts Service). The results represent a pooling of 3 musk samples each. The compounds in the table are listed by their retention time.

**Table 2:** Phylum-level assignments of operational taxonomic units (OTUs) in the musk pods of unmated and mated Chinese forest musk deer males.

Class	Phylum	Number of OTUs		Percentage out of total number of OTUs (%)		Number of reads		Percentage out of total number of reads (%)	
		MM	UM	MM	UM	MM	UM	MM	UM
Bacteria	Proteobacteria	351.6 ± 40.7^a^	133.2 ± 8.9^b^	32.73^a^	23.19^b^	1948.6 ± 223.6^a^	774.6 ± 137.3^b^	29.44^a^	11.88^b^
	Bacteroidetes	74.6 ± 9.7	52.2 ± 6.4	6.94	9.12	118.6 ± 20.8	330.8 ± 84.7	1.72^a^	4.92^b^
	Firmicutes	86.2 ± 5.4^a^	167 ± 13.8^b^	8.26^a^	29.11^b^	359.0 ± 39.3^a^	2112.6 ± 388.4^b^	5.45^a^	32.32^b^
	Actinobacteria	52.2 ± 9.6	58.2 ± 3.2	4.72^a^	10.14^b^	362.4 ± 38.6^a^	1720.0 ± 280.2^b^	5.50^a^	26.63^b^
	Fusobacteria	6.2 ± 0.7	5.4 ± 0.4	0.62	0.94	34.2 ± 9.1^a^	996.6 ± 228.7^b^	0.47^a^	14.65^b^
	OD1	3.6 ± 1.3	1.0 ± 0.3	0.4	0.17	4.4 ± 1.8	2.2 ± 1.0	0.08	0.03
	WS3	1 ± 0.5	0.2 ± 0.2	0.08	0.03	1.4 ± 0.9	0.2 ± 0.2	0.02	0.00
	Cyanobacteria	5.2 ± 0.7	6.8 ± 0.9	0.50^a^	1.18^b^	10.2 ± 2.2	10.6 ± 1.4	0.14	0.18
	Verrucomicrobia	8 ± 2.2	2.8 ± 0.6	0.7	0.48	16.2 ± 4.2	5.8 ± 1.3	0.24	0.11
	Tenericutes	3.6 ± 0.5	2.8 ± 0.5	0.35	0.49	55.2 ± 9.9	18.6 ± 6.5	0.85	0.36
	Synergistetes	1.4 ± 0.2	1.2 ± 0.5	0.14	0.22	1.6 ± 0.4	1.6 ± 0.7	0.02	0.02
	Spirochaetes	0.4 ± 0.2	0.2 ± 0.2	0.04	0.03	0.6 ± 0.4	0.2 ± 0.2	0.01	0.00
	TM7	8.6 ± 1.5^a^	2.6 ± 0.7^b^	0.77	0.44	15.2 ± 3.8	3.6 ± 1.3	0.21^a^	0.06^b^
	Planctomycetes	13.8 ± 2^a^	4.6 ± 0.4^b^	1.29^a^	0.81^b^	19 ± 2.7^a^	5.4 ± 0.8^b^	0.28^a^	0.10^b^
	Nitrospira	3.2 ± 1.2	1.4 ± 0.4	0.24	0.25	5.6 ± 1.9	1.4 ± 0.4	0.08	0.03
	Gemmatimonadetes	20.2 ± 3.3	9.8 ± 2.1	1.84	1.73	29.4 ± 5.9	11.2 ± 2.3	0.42	0.22
	Fibrobacteres	0.4 ± 0.2	0.0 ± 0.0	0.04	0	0.6 ± 0.4	0.0 ± 0.0	0.01	0.00
	Elusimicrobia	1.4 ± 0.7	0.0 ± 0.0	0.11	0	1.6 ± 0.8	0.0 ± 0.0	0.02	0.00
	Deinococcus-Thermus	1 ± 0.3	1.4 ± 0.5	0.09	0.25	1.6 ± 0.5	1.8 ± 0.7	0.02	0.03
	Deferribacteres	0.2 ± 0.2	0.0 ± 0.0	0.01	0	0.2 ± 0.2	0.0 ± 0.0	0.00	0.00
	Chrysiogenetes	0.4 ± 0.2	0.2 ± 0.2	0.03	0.03	0.8 ± 0.6	0.2 ± 0.2	0.01	0.00
	Chloroflexi	10.8 ± 2.1^a^	2.6 ± 0.2^b^	0.98	0.45	14.0 ± 3.4	2.6 ± 0.2	0.20^a^	0.05^b^
	Chlorobi	2 ± 0.6	0.4 ± 0.2	0.17	0.07	10.2 ± 1.4^a^	0.6 ± 0.4^b^	0.16^a^	0.01^b^
	Chlamydiae	2.4 ± 0.5	0.6 ± 0.2	0.24	0.1	3.6 ± 0.6	1 ± 0.6	0.06	0.02
	Caldiserica	0.8 ± 0.2^a^	0.0 ± 0.0 ^b^	0.07^a^	0.00^b^	0.8 ± 0.2^a^	0.0 ± 0.0^b^	0.01^a^	0.00^b^
	Armatimonadetes	0.4 ± 0.2	0.6 ± 0.2	0.03	0.11	0.4 ± 0.2	0.6 ± 0.2	0.00	0.01
	Acidobacteria	54.8 ± 14.3	16.8 ± 2.8	4.73	2.95	73.2 ± 20.6	21.4 ± 2.9	1.00	0.39
	Bacteria unknown	39.2 ± 7.1^a^	11.4 ± 1.6^b^	3.53^a^	1.98^b^	46.6 ± 8.2^a^	12.8 ± 1.8^b^	0.68^a^	0.23^b^
Archaea	Euryarchaeota	318.8 ± 35.3^a^	88.2 ± 20.1^b^	29.81^a^	15.19^b^	3779.2 ± 850.7^a^	414.4 ± 120.5^b^	52.67^a^	7.67^b^
	Crenarchaeota	3.6 ± 0.4	2.4 ± 0.2	0.36	0.41	14.4 ± 5.6	4.2 ± 1.2	0.19	0.07
	Archaea unknown	1.6 ± 0.4	0.4 ± 0.2	0.15	0.06	2.0 ± 0.6	1.0 ± 0.6	0.03	0.01
Unknown	Unknown	0.6 ± 0.4	0.2 ± 0.2	0.03	0.07	0.8 ± 0.6	0.2 ± 0.2	0.01	0.00

Note: After Bonferroni correction, reads number and OTU number are represented as least squares means ± standard deviation. Associations between mating status and phylum-level assignments were assessed using the least-squares method (GLM procedure, SAS version 8.02, 2001). Significance of the least squares means was tested with the Tukey’s Multiple Range test. Least squares means in a line with different superscripts are significantly (^a,b^*P* < 0.01) different. Percentage of OTU number and percentage of reads number are represented as median values. Median percentage values of bacterial and archaea phyla in samples from mated and unmated males found by using the RDP Classifier. Significance of the median percentage values was tested with the Mann-Whitney *U* test (^a,b^*P* < 0.01). More than 95% of the sequences in all samples were found to belong to the six most populated underlined phyla.

## References

[b1] He L. *et al.* Effects of crowding and sex on fecal cortisol levels of captive forest musk deer. Biological research 47, 48, 10.1186/0717-6287-47-48 (2014). 25418206 PMC4222733

[b2] Wang Y. H. & Moschus berezovskii R. B. The IUCN Red List of Threatened Species. Version 2014.3. Downloaded on 30 December 2014 (2008), http://www.iucnredlist.org/details/13894/0.

[b3] Green M. The distribution, status and conservation of the Himalayan musk deer Moschus chrysogaster. Biological Conservation 35, 347–375, 10.1016/0006-3207(86)90094-7 (1986).

[b4] Green M. J. Scent-marking in the Himalayan musk deer (Moschus chrysogaster). Journal of Zoology 1, 721–737 (1987).

[b5] Feng W., You Y., Yong H., Li G. & Gu D. Histological observation of Chinese forest musk deer (Moschus berezovskii Flerov) musk glands (in Chinese). The Zoological Journal, 13–15 (1981).

[b6] Hawkins T. H. Musk and the musk deer. Nature 166, 262 (1950). 15439270 10.1038/166262a0

[b7] Sokolov V. E., Kagan M. Z., Vasilieva V. S., Prihodko V. I. & Zinkevich E. P. Musk deer (Moschus moschiferus): Reinvestigation of main lipid components from preputial gland secretion. Journal of chemical ecology 13, 71–83, 10.1007/BF01020352 (1987). 24301360

[b8] Gilbert S. F., Sapp J. & Tauber A. I. A symbiotic view of life: We have never been individuals. The Quarterly review of biology 87, 325–341 (2012). 23397797 10.1086/668166

[b9] McFall-Ngai M. *et al.* Animals in a bacterial world, a new imperative for the life sciences. Proceedings of the National Academy of Sciences 110, 3229–3236 (2013). 10.1073/pnas.1218525110 PMC3587249 23391737

[b10] McCune B., Grace J. B. & Urban D. L. Analysis of ecological communities. Vol. 28 (MjM software design Gleneden Beach, OR, 2002).

[b11] Lee Z. M.-P., Bussema C. & Schmidt T. M. rrnDB: documenting the number of rRNA and tRNA genes in bacteria and archaea. Nucleic acids research 37, D489–D493 (2009). 18948294 10.1093/nar/gkn689 PMC2686494

[b12] Theis K. R. *et al.* Symbiotic bacteria appear to mediate hyena social odors. Proceedings of the National Academy of Sciences 110, 19832–19837 (2013). 10.1073/pnas.1306477110 PMC3856825 24218592

[b13] Theimer E. T. Fragrance Chemistry: The Science of the Sense of Smell. Ch. 12, 433 (Mookherjee, B. D. & Wilson, R. A.) The chemistry and fragrance of natural musk compounds (2012).

[b14] Crawford P. A. *et al.* Regulation of myocardial ketone body metabolism by the gut microbiota during nutrient deprivation. Proceedings of the National Academy of Sciences 106, 11276–11281 (2009). 10.1073/pnas.0902366106 PMC2700149 19549860

[b15] Mount D. W. Using the Basic Local Alignment Search Tool (BLAST). CSH protocols 2007, pdb top17, 10.1101/pdb.top17 (2007). 21357135

[b16] Parks D. H. & Beiko R. G. Measuring community similarity with phylogenetic networks. Mol Biol Evol 29, 3947–3958, 10.1093/molbev/mss200 (2012). 22915830

[b17] Huson D. H. & Bryant D. Application of phylogenetic networks in evolutionary studies. Mol Biol Evol 23, 254–267, 10.1093/molbev/msj030 (2006). 16221896

[b18] Bryant D. & Moulton V. Neighbor-net: an agglomerative method for the construction of phylogenetic networks. Mol Biol Evol 21, 255–265, 10.1093/molbev/msh018 (2004). 14660700

[b19] Angen O., Mutters R., Caugant D. A., Olsen J. E. & Bisgaard M. Taxonomic relationships of the [Pasteurella] haemolytica complex as evaluated by DNA-DNA hybridizations and 16S rRNA sequencing with proposal of Mannheimia haemolytica gen. nov., comb. nov., Mannheimia granulomatis comb. nov., Mannheimia glucosida sp. nov., Mannheimia ruminalis sp. nov. and Mannheimia varigena sp. nov. International journal of systematic bacteriology 49 Pt 1, 67–86 (1999). 10028248 10.1099/00207713-49-1-67

[b20] Angen O., Ahrens P. & Bisgaard M. Phenotypic and genotypic characterization of Mannheimia (Pasteurella) haemolytica-like strains isolated from diseased animals in Denmark. Veterinary microbiology 84, 103–114 (2002). 11731163 10.1016/s0378-1135(01)00439-4

[b21] Do J., Kitatsuji E. & Yoshii E. Study on the components of musk. I. Ether soluble components. Chemical and Pharmaceutical Bulletin 23, 629–635 (1975).

[b22] Altschul S. F., Gish W., Miller W., Myers E. W. & Lipman D. J. Basic local alignment search tool. J Mol Biol 215, 403–410, 10.1016/S0022-2836(05)80360-2 (1990). 2231712

[b23] Jackman P. & Noble W. Normal axillary skin in various populations. Clinical and experimental dermatology 8, 259–268 (1983). 6411399 10.1111/j.1365-2230.1983.tb01778.x

[b24] Sanford J. A. & Gallo R. L. Functions of the skin microbiota in health and disease. Seminars in immunology 25, 370–377, 10.1016/j.smim.2013.09.005 (2013). 24268438 PMC4219649

[b25] Zeng B. *et al.* The bacterial communities associated with fecal types and body weight of rex rabbits. Scientific reports 5, 10.1038/srep09342 (2015). PMC4366860 25791609

[b26] Yan Y. A preliminary study on the factors affecting musk production in Siberian musk deer (in Chinese). Journal of Chinese Medicinal Materials 2, 11–13 (1985).

[b27] Caporaso J. G. *et al.* QIIME allows analysis of high-throughput community sequencing data. Nature methods 7, 335–336 (2010). 20383131 10.1038/nmeth.f.303 PMC3156573

[b28] Edgar R. C., Haas B. J., Clemente J. C., Quince C. & Knight R. UCHIME improves sensitivity and speed of chimera detection. Bioinformatics 27, 2194–2200, 10.1093/bioinformatics/btr381 (2011). 21700674 PMC3150044

[b29] Matsuda F., Tsugawa H. & Fukusaki E. Method for assessing the statistical significance of mass spectral similarities using basic local alignment search tool statistics. Analytical chemistry 85, 8291–8297, 10.1021/ac401564v (2013). 23944154

[b30] Wang Q., Garrity G. M., Tiedje J. M. & Cole J. R. Naive Bayesian classifier for rapid assignment of rRNA sequences into the new bacterial taxonomy. Applied and environmental microbiology 73, 5261–5267 (2007). 17586664 10.1128/AEM.00062-07 PMC1950982

[b31] Garrity G. M. *et al.* Introduction to the Taxonomic Outline of Bacteria and Archaea (TOBA) Release 7.7. The Taxonomic Outline of Bacteria and Archaea 7, 1–5 (2007).

[b32] Schloss P. D. *et al.* Introducing mothur: open-source, platform-independent, community-supported software for describing and comparing microbial communities. Applied and environmental microbiology 75, 7537–7541 (2009). 19801464 10.1128/AEM.01541-09 PMC2786419

[b33] Langille M. G. *et al.* Predictive functional profiling of microbial communities using 16S rRNA marker gene sequences. Nature biotechnology 31, 814–821 (2013). 10.1038/nbt.2676 PMC3819121 23975157

