# Supplementary material for: The musk chemical composition and microbiota of Chinese forest musk deer males
Source: Sci Rep. 2016 Jan 8;6:18975. doi: 10.1038/srep18975 (PMC4705530; doi:10.1038/srep18975)
Supplement: Supplementary Information [file srep18975-s1.doc]

**Supporting Information**

The musk chemical composition and microbiota of Chinese forest musk deer males

Diyan Li^1†^, Binlong Chen^1†^, Long Zhang^1†^, Uma Gaur^1^, Tianyuan Ma^1^, Hang Jie^2^, Guijun Zhao^2^, Nan Wu^1^, Zhongxian Xu^1^, Huailiang Xu^1^, Yongfang Yao^1^, Ting Lian^1^, Xiaolan Fan^1^, Deying Yang^1^, Mingyao Yang^1^, Qing Zhu^1*^ and Jessica Satkoski Trask^3*^

^1^ Farm Animal Genetic Resources Exploration and Innovation Key Laboratory of Sichuan Province, Sichuan Agricultural University, Chengdu, P.R.China, 61130;

^2^ Laboratory of Medicinal Animal, Chongqing Institute of Medicinal Plant Cultivation, Nanchuan, Chongqing, P.R.China, 408435;

^3^ Department of Anthropology and California National Primate Research Center, University of California, Davis, Davis, California.

^†^ These authors contributed equally to this work.

* Author to whom correspondence should be addressed; E-Mail: [zhuqingsicau@163.com](mailto:qingzhusicau@163.com); jsatkosk@gmail.com

**TableS1.** Musk production record by mated and unmated males of Chinese forest musk deer

|  | Sample name | Musk weight(g) | Father/  Mother | Born data | Behavioral observation time | Musk collection time | Barcode | Clean number of sequences |
| --- | --- | --- | --- | --- | --- | --- | --- | --- |
| Mated males (MM) | MM1 | 2.311 | 53/47 | 5/01/2011 | Late Nov 2013 | 10/09/2014 | GACATGT | 12630 |
|  | MM2 | 0.534 | 50/48 | 5/06/2010 | Late Nov 2013 | 10/09/2014 | GATGTAC | 15008 |
|  | MM3 | 0.422 | 53/59 | 5/12/2011 | Late Nov 2013 | 10/09/2014 | CTCACGC | 18590 |
|  | MM4 | 0.955 | 53/62 | 5/03/2011 | Late Nov 2013 | 10/09/2014 | CGAGAGA | 17941 |
|  | MM5 | 1.368 | 50/63 | 5/05/2010 | Late Nov 2013 | 10/09/2014 | GAGATAT | 13695 |
| Mean ± SE | - | 1.12±0.34 | - | - | - | - | - | 15572.80±1166.56 |
| Unmated males (UM) | UM1 | 30.453 | 53/47 | 5/02/2011 | Late Nov 2013 | 10/09/2014 | GTCGATC | 16484 |
|  | UM2 | 18.537 | 50/59 | 5/08/2011 | Late Nov 2013 | 10/09/2014 | GCGTGAT | 13315 |
|  | UM3 | 12.689 | 53/46 | 5/11/2010 | Late Nov 2013 | 10/09/2014 | CATCATC | 18832 |
|  | UM4 | 20.715 | 53/63 | 5/06/2011 | Late Nov 2013 | 10/09/2014 | GCGCGCA | 7573 |
|  | UM5 | 31.532 | 53/64 | 5/07/2011 | Late Nov 2013 | 10/09/2014 | TGTCTCG | 8111 |
| Mean ± SE | - | 22.79±3.6 | - | - | - | - | - | 12863.00 ±2230.59 |

**Figure S1.** The sexually mature male is armed with two abnormally long teeth is noted by the red ellipse with dash line. The photograph of the deer has been obtained from farm of Chongqing Institute of Medicinal Plant Cultivation. Author Hang Jie of this manuscript took the photograph.


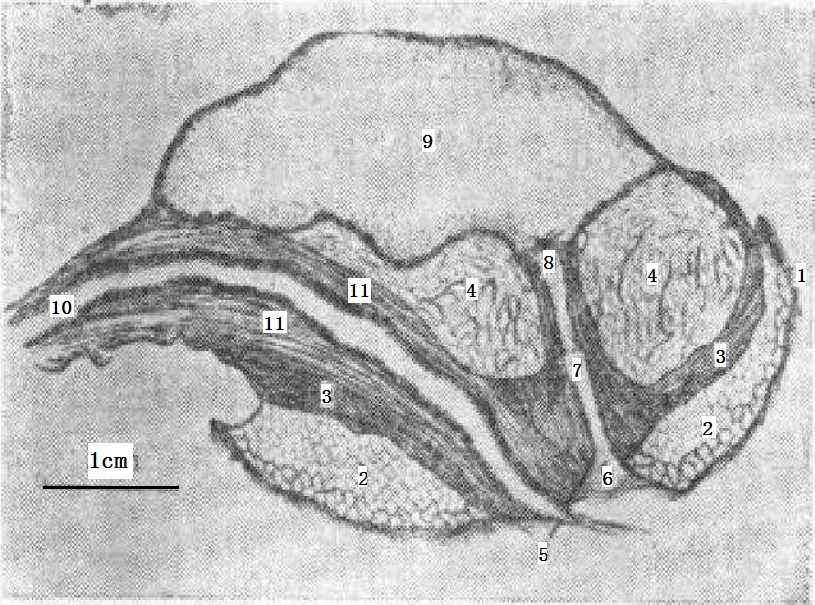


**Figure S2.** Musk pod and generative organs of the male Musk Deer. The drawing has been obtained from a previous published article in Chinese by Feng et al (1981).

Note：1. Skin 2. Sweat gland and sebaceous gland 3. Striated muscle 4. Preputial gland 5.Preputial orifice 6. Orifice of the musk sac 7. Ductusexcretorius of musk 8. Musk pod neck 9. Musk pod 10. Penis 11. Annulus praeputialis [^1^](#_ENREF_1).

1 Feng, W., You, Y., Yong, H., Li, G. & Gu, D. Histological observation of Chinese forest musk deer (Moschus berezovskii Flerov) musk glands (in Chinese). *The Zoological Journal*, 13-15 (1981).

**Figure S3**. Rarefaction analysis for mated and unmated musk samples based on MOTHUR program. Sample-based rarefaction curve showing the increase in Richness (A), ACE index (B), Chao1 index (C), Shannon index (D) as a function of the sequence number of individuals sampled.


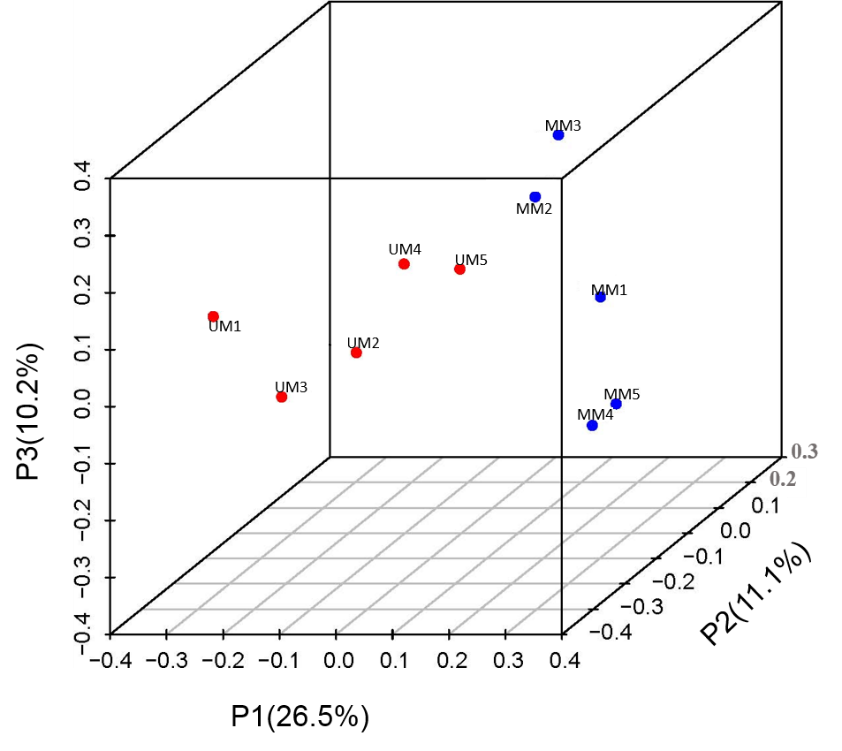


**Figure S4.** Principal coordinate (PCoA) analysis of the community structure using UniFrac distances. Red and blue circles represent the musk microbiotas from the unmated and mated Chinese forest musk deer males, respectively. Distances between circles on the ordination plot reflect relative dissimilarities in community structures.

**Figure S5**. Breeding yard for Chinese forest musk deer males. One to three represent small living rooms, playground and musk deer respectively. The photograph of the deer has been obtained from farm of Chongqing Institute of Medicinal Plant Cultivation. Author Hang Jie of this manuscript took the photograph.
